# Supplementary material for: Galleria mellonella immune melanization is fungicidal during infection
Source: Commun Biol. 2022 Dec 12;5:1364. doi: 10.1038/s42003-022-04340-6 (PMC9744840; doi:10.1038/s42003-022-04340-6)
Supplement: Supplementary file 2 — Supplementary Information [file 42003_2022_4340_MOESM2_ESM.pdf]

Supplementary Information

Galleria mellonella immune melanization is fungicidal during infection

Daniel F. Q. Smith[1], Quigly Dragotakes[1], Madhura Kulkarni[1], J. Marie Hardwick[1], Arturo Casadevall*[1]

[1] W. Harry Feinstone Department of Molecular Microbiology and Immunology, The Johns Hopkins Bloomberg School of Public Health, Baltimore, MD 21205, United States

*Corresponding Author: acasade1@jh.edu

Supplementary Figure 1: Fungal melanization does not affect GFP signal in the H99-GFP strain.

Supplementary Figure 2: Dissection of *C. albicans* infected *G. mellonella*

Supplementary Figure 3: Summary figure of methods used

List of Supplementary Movies 1-12

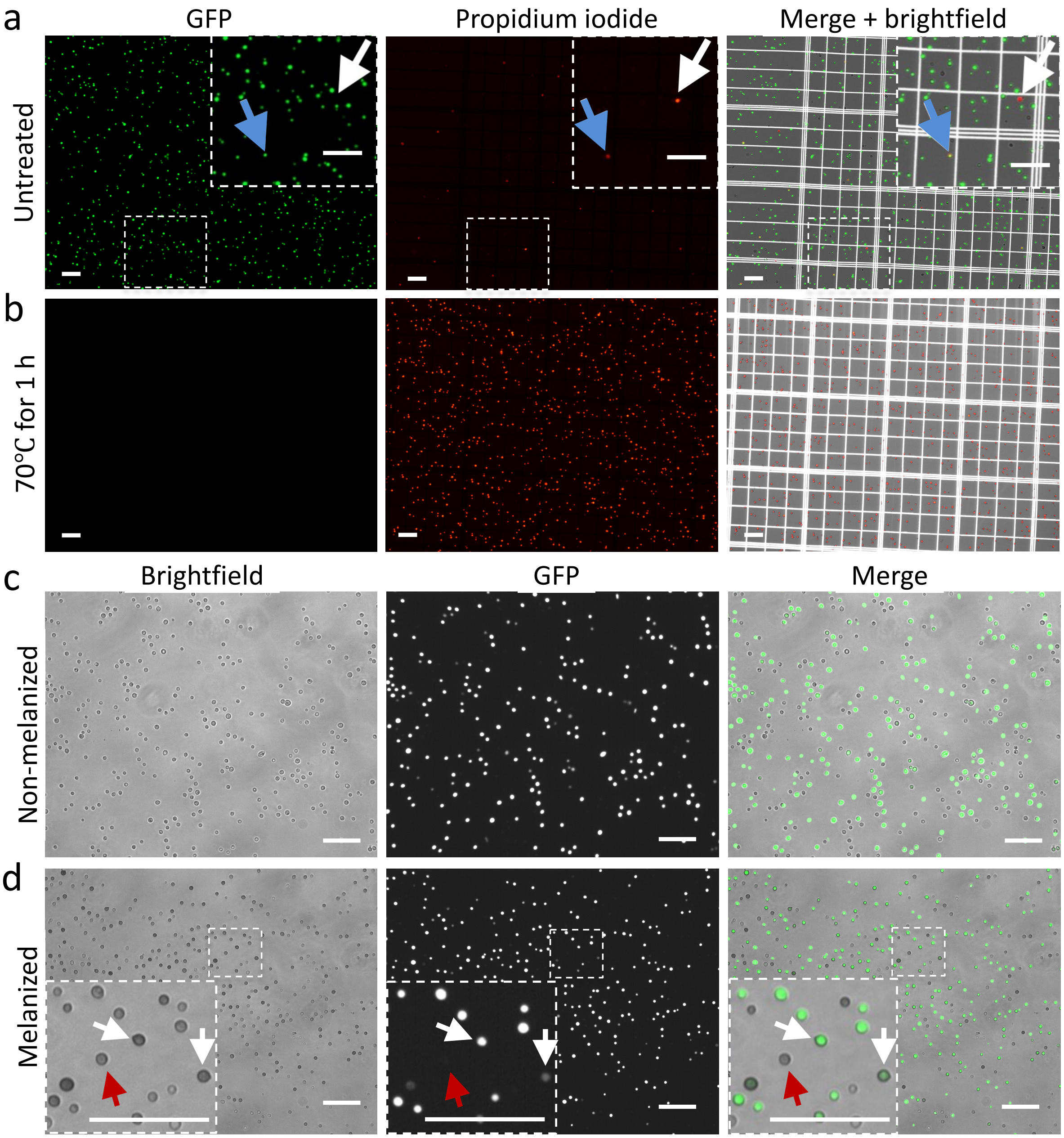

Supplementary Figure S1. Fungal melanization does not affect GFP signal in the H99-GFP strain. (A-B) GFP signal is lost upon heat treatment for 1 hour, and cells become exclusively propidium iodine positive Insets in (A) indicate representative images of cells in untreated condition, with the white arrows pointing to a PI-only positive cell, and a blue arrow pointing to a PI and GFP double positive cell. Non-melanized H99-GFP (C) and melanized H99-GFP (D) have comparable levels of GFP-fluorescence. Insets in (D) indicate representative selections of melanized cells that have GFP signal (white arrows) and melanized cells that lack GFP signal (red arrow). Scale bars represent 50 µm

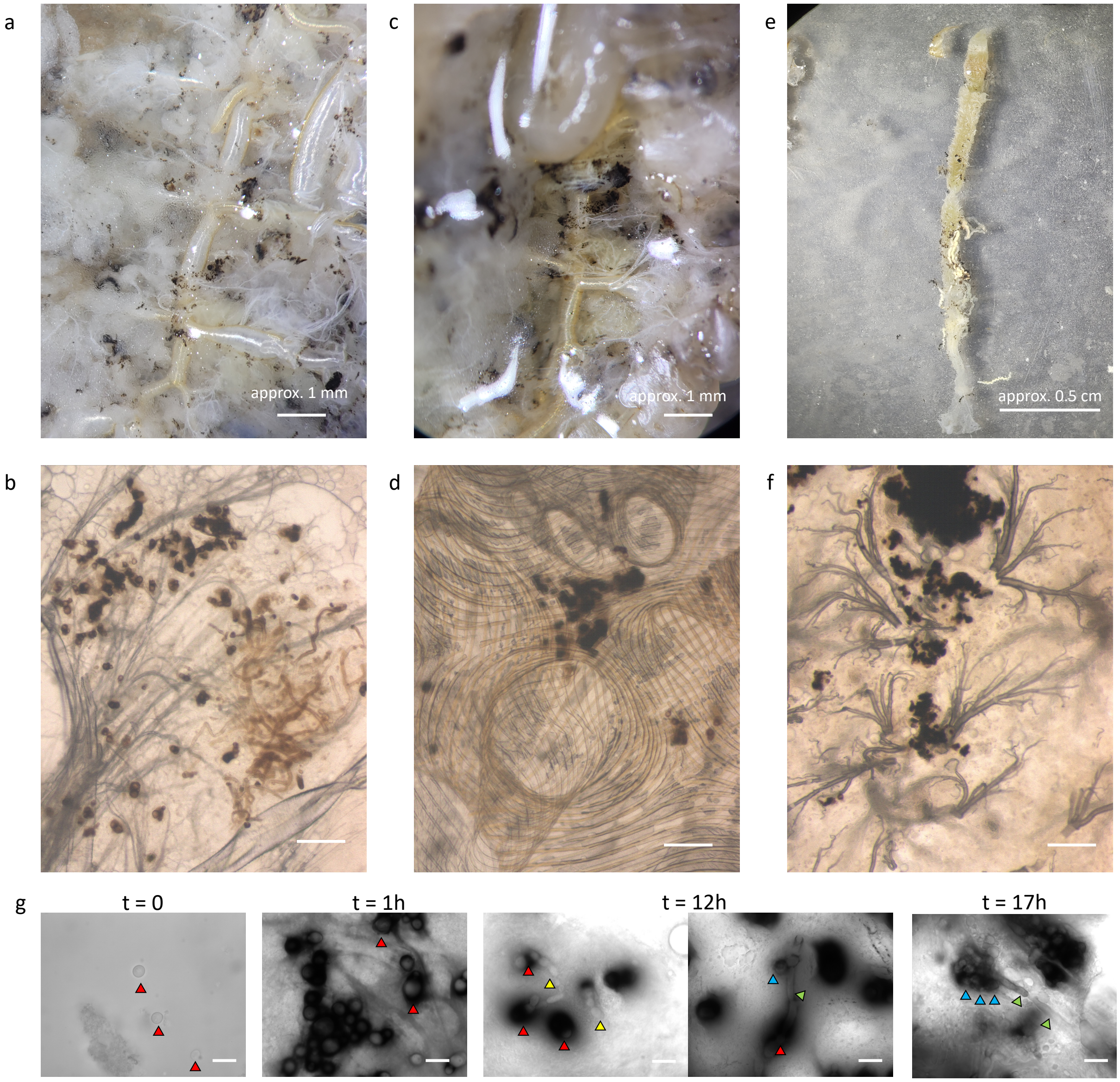

Supplementary Figure S2. Dissection of *C. albicans* infected *G. mellonella*. There does not appear to be any specific tissue tropism for *C. albicans* infections in *G. mellonella,* with visible melanized nodules found in scattered in the Fat Bodies (A-C red arrow), trachea (A,C,D white arrow), and the gut (E,F). Microscopic analysis of the dissected tissue also reveals hyphal structures with reduced pigmentation compared to the yeast-like structures (B). (G) A similar progression of *C. albicans* morphological progression is seen in dissected tissues infections as is seen during the *in vitro* microscopy. At 0 minutes, there is no melanin encapsulation of the *C. albicans* yeast, whereas after 1 h, there is extensive melanization of *C. albicans* yeast. At 12 h, non-melanized hyphal and pseudohyphal structures are visible along with melanized yeast and potential laterally budding blastoconidium. By 17 h, there appears to be melanized laterally-budded blastoconidium with non-melanized hyphae, similar to what we see between hour 12 and 16 in the timelapse microscopy. Red arrows in (G) indicate yeast, yellow arrows indicate pseudohyphae, green arrows indicate hyphae, and blue arrows indicate potential laterally-budded blastoconidium. Scale bars in (A,C) represent approximately 1 mm, in (E) represents approximately 0.5 cm, in (B, D, F) represent 50 µm, and in (G) represent 10 µm.

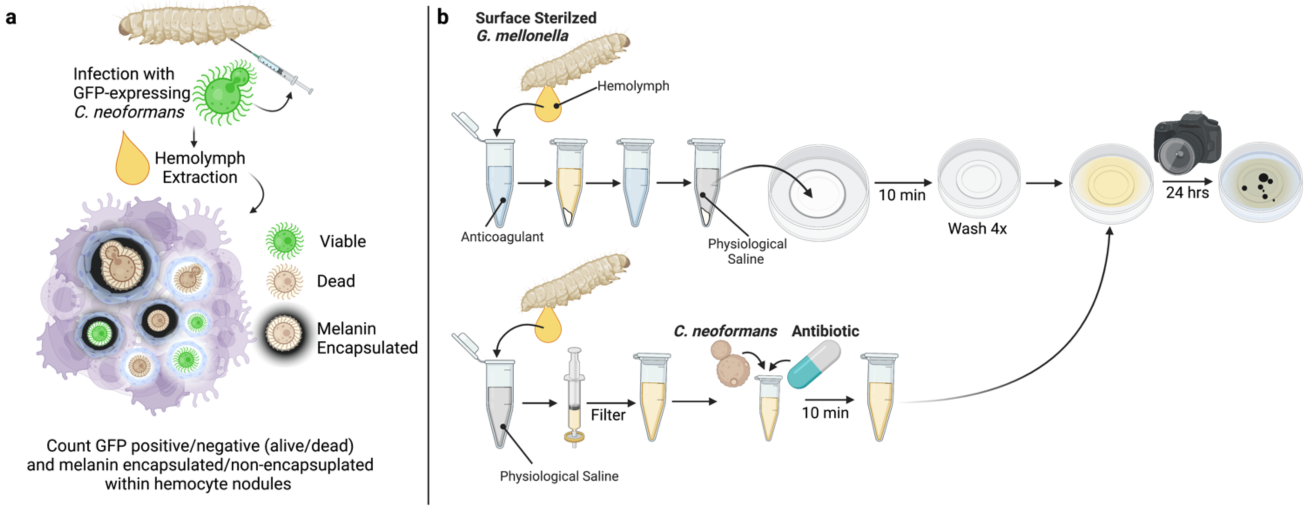

Supplementary Figure S3. Summary Figure of methods used. *(*A) We assayed fungal viability within nodules by infecting larvae with a GFP-expressing strain of *C. neoformans.* We extracted the infected hemolymph and imaged the nodules under a microscope and tallied GFP signal status (positive/negative) and melanin-encapsulation status (melanin-encapsulated or not melanin-encapsulated). (B) For the *in vitro* timelapse microscopy experiments, first, hemolymph from surface sterilized larvae was collected into anticoagulation buffer, hemocytes were centrifuged, washed in anticoagulant, and resuspended in insect physiological saline, and left to settle on a MatTek dish for 10 minutes. Simultaneously, hemolymph was collected into insect physiological saline, filtered with a PVDF 0.22 µm filter. Antibiotic and fungus were then added, left to sit for 10 minutes, and added to the adherent hemocytes following 4 washes with insect physiological saline. Timelapse microscopy was then performed for up to 24 hours.

List of Supplementary Movies 1-12

Supplementary Movie 1_C. neoformans timelapse

Supplementary Movie 2_C. neoformans timelapse

Supplementary Movie 3_Melanin Ghost vs heat killed

Supplementary Movie 4 _Melanin ghost without hemocytes

Supplementary Movie 5_Melanin ghost timelapse

Supplementary Movie 6_Hemocyte-ghost interactions

Supplementary Movie 7_In situ nodule projection

Supplementary Movie 8_Melanin Bloom Candida

Supplementary Movie 9_Candida albicans escape

Supplementary Movie 10_Candida auris pseudohyphae

Supplementary Movie 11_C. neoformans Anticoagulation Buffer

Supplementary Movie 12_No fungus timelapse
