# Supplementary material for: Galleria mellonella immune melanization is fungicidal during infection
Source: Commun Biol. 2022 Dec 12;5:1364. doi: 10.1038/s42003-022-04340-6 (PMC9744840; doi:10.1038/s42003-022-04340-6)
Supplement: Supplementary file 3 — Description of Additional Supplementary Files [file 42003_2022_4340_MOESM3_ESM.pdf]

Description of Additional Supplementary Files

Filename: Supplementary Movie 1_C. neoformans timelapse
Description: Melanization response in hemolymph in response to *C. neoformans.*

Filename: Supplementary Movie 2_C. neoformans timelapse
Description: Melanization response in hemolymph in response to *C. neoformans*

Filename: Supplementary Movie 3_Melanin Ghost vs heat killed
Description: Melanization response in hemolymph in response to heat-killed *C. neoformans* and isolated fungal melanins.

Filename: Supplementary Movie 4_Melanin ghost without hemocytes
Description: Melanization response in hemolymph in response to isolated fungal melanins.

Filename: Supplementary Movie 5_ Melanin ghost timelapse
Description: Melanization response in hemolymph in response to isolated fungal melanin.

Filename: Supplementary Movie 6_ Hemocyte-ghost interactions
Description: Timelapse of the interactions between the insect hemocytes and isolated fungal melanins

Filename: Supplementary Movie 7_ In situ nodule projection
Description: 3D Rendering of a fungal nodule within *G. mellonella* using different Z-stacked microscopy images.

Filename: Supplementary Movie 1_C. neoformans timelapse

Description: Melanization response in hemolymph in response to C. neoformans.

Filename: Supplementary Movie 2_C. neoformans timelapse

Description: Melanization response in hemolymph in response to C. neoformans.

Filename: Supplementary Movie 3_Melanin Ghost vs heat killed

Description: Melanization response in hemolymph in response to heat-killed C. neoformans and isolated fungal melanins.

Filename: Supplementary Movie 4_Melanin ghost without hemocytes

Description: Melanization response in hemolymph in response to isolated fungal melanins.

Filename: Supplementary Movie 5_ Melanin ghost timelapse

Description: Melanization response in hemolymph in response to isolated fungal melanin.

Filename: Supplementary Movie 6_ Hemocyte-ghost interactions

Description: Timelapse of the interactions between the insect hemocytes and isolated fungal melanins

Filename: Supplementary Movie 7_ In situ nodule projection

Description: 3D Rendering of a fungal nodule within G. mellonella using different Z-stacked microscopy images.

Filename: Supplementary Movie 8_ Melanin Bloom Candida

Description: Melanization response in hemolymph in response to C. albicans yeast and hyphal forms.

Filename: Supplementary Movie 9_ Candida albicans escape

Description: Examples of C. albicans surviving and growing through immune melanin-encapsulation.

Filename: Supplementary Movie 10_ Candida auris pseudohyphae.

Description: Melanization response in hemolymph in response to C. auris yeast and pseudohyphal forms.

Filename: Supplementary Movie 11_ C. neoformans Anticoagulation Buffer

Description: Timelapse of hemocytes and hemolymph interacting with C. neoformans in an anticoagulation buffer to prevent coagulation and melanization.

Filename: Supplementary Movie 12_ No fungus timelapse

Description: Timelapse of hemocytes and hemolymph with no fungus or fungal component added.
