# Supplementary material for: Galleria mellonella immune melanization is fungicidal during infection
Source: Commun Biol. 2022 Dec 12;5:1364. doi: 10.1038/s42003-022-04340-6 (PMC9744840; doi:10.1038/s42003-022-04340-6)
Supplement: Supplementary file 5 — Reporting Summary [file 42003_2022_4340_MOESM5_ESM.pdf]

nature portfolio

Corresponding author(s): Daniel F. Q. Smith

Last updated by author(s): Oct 13, 2022

Reporting Summary

Nature Portfolio wishes to improve the reproducibility of the work that we publish. This form provides structure for consistency and transparency in reporting. For further information on Nature Portfolio policies, see our Editorial Policies and the Editorial Policy Checklist.

Statistics

For all statistical analyses, confirm that the following items are present in the figure legend, table legend, main text, or Methods section.

n/a	Confirmed	
☐	☒	The exact sample size (n) for each experimental group/condition, given as a discrete number and unit of measurement
☐	☒	A statement on whether measurements were taken from distinct samples or whether the same sample was measured repeatedly
☐	☒	The statistical test(s) used AND whether they are one- or two-sided<br>*Only common tests should be described solely by name; describe more complex techniques in the Methods section.*
☒	☐	A description of all covariates tested
☒	☐	A description of any assumptions or corrections, such as tests of normality and adjustment for multiple comparisons
☐	☒	A full description of the statistical parameters including central tendency (e.g. means) or other basic estimates (e.g. regression coefficient) AND variation (e.g. standard deviation) or associated estimates of uncertainty (e.g. confidence intervals)
☐	☒	For null hypothesis testing, the test statistic (e.g. F, t, r) with confidence intervals, effect sizes, degrees of freedom and P value noted<br>*Give P values as exact values whenever suitable.*
☒	☐	For Bayesian analysis, information on the choice of priors and Markov chain Monte Carlo settings
☒	☐	For hierarchical and complex designs, identification of the appropriate level for tests and full reporting of outcomes
☒	☐	Estimates of effect sizes (e.g. Cohen's d, Pearson's r), indicating how they were calculated

Our web collection on statistics for biologists contains articles on many of the points above.

Software and code

Policy information about availability of computer code

Data collection	Timelapse microscopy data was collected using the open source OpenFlexure software, code, and 3D-printed microscope as described in the paper. A link to the software is found here: https://openflexure.org/software/raspbian-openflexure/
Data analysis	Data was graphed and statistical tests were done using Prism GraphPad Version 9. Timelapse microscopy movies were analyzed using open source FIJI (Version 2.1.0) as described in the text. FIJI can be downloaded using this link: https://imagej.net/software/fiji/

For manuscripts utilizing custom algorithms or software that are central to the research but not yet described in published literature, software must be made available to editors and reviewers. We strongly encourage code deposition in a community repository (e.g. GitHub). See the Nature Portfolio guidelines for submitting code & software for further information.

Data

Policy information about availability of data

All manuscripts must include a data availability statement. This statement should provide the following information, where applicable:

- Accession codes, unique identifiers, or web links for publicly available datasets
- A description of any restrictions on data availability
- For clinical datasets or third party data, please ensure that the statement adheres to our policy

The datasets generated during and/or analyzed during the current study are available in the Figshare repository using the following link: https://figshare.com/projects/Galleria_mellonella_immune_melanization_is_fungicidal_during_infection/138123

Field-specific reporting

Please select the one below that is the best fit for your research. If you are not sure, read the appropriate sections before making your selection.

☒ Life sciences ☐ Behavioural & social sciences ☐ Ecological, evolutionary & environmental sciences

For a reference copy of the document with all sections, see nature.com/documents/nr-reporting-summary-flat.pdf

Life sciences study design

All studies must disclose on these points even when the disclosure is negative.

Sample size	No sample size calculations were done. Most experiments were performed in biological triplicate.
Data exclusions	For the timelapse microscopy data, movies were excluded in the event of bacterial contamination or equipment error (such as freezing or crashing of the microscope, resulting in unusable videos)
Replication	All replicates are reported, and reproducible. Galleria mellonella larvae exhibit large degrees of variability between orders since there is no isogenic/inbred strain of G. mellonella. As a result, there is a large degree of variability between biological replicates for the timelapse movies. For this reason, we normalized the data to the control group within each biological replicate.
Randomization	Healthy Galleria mellonella larvae were chosen at random for infection or hemolymph extraction before and after infection.
Blinding	Blinding was not done for this project.

Reporting for specific materials, systems and methods

We require information from authors about some types of materials, experimental systems and methods used in many studies. Here, indicate whether each material, system or method listed is relevant to your study. If you are not sure if a list item applies to your research, read the appropriate section before selecting a response.

Materials & experimental systems

n/a	Involved in the study	
☒	☐	Antibodies
☒	☐	Eukaryotic cell lines
☒	☐	Palaeontology and archaeology
☐	☒	Animals and other organisms
☒	☐	Human research participants
☒	☐	Clinical data
☒	☐	Dual use research of concern

Methods

n/a	Involved in the study	
☒	☐	ChIP-seq
☒	☐	Flow cytometry
☒	☐	MRI-based neuroimaging

Animals and other organisms

Policy information about studies involving animals; ARRIVE guidelines recommended for reporting animal research

Laboratory animals	Galleria mellonella Wax Moth
Wild animals	None
Field-collected samples	None
Ethics oversight	Galleria mellonella do not require ethics oversight as invertebrate model organisms.

Note that full information on the approval of the study protocol must also be provided in the manuscript.
