# Supplementary material for: Galleria mellonella immune melanization is fungicidal during infection
Source: Commun Biol. 2022 Dec 12;5:1364. doi: 10.1038/s42003-022-04340-6 (PMC9744840; doi:10.1038/s42003-022-04340-6)
Supplement: Supplementary file 4 — Supplementary Movies 1-12 [file 42003_2022_4340_MOESM4_ESM.zip › Supplementary Movie Captions.docx]

**Supplementary Movie Descriptions.**

**Supplementary Movie 1.** Timelapse of the *in vitro* melanization response in hemolymph in response to *C. neoformans.*

**Supplementary Movie 2.** Timelapse of the *in vitro* melanization response in hemolymph in response to *C. neoformans.*

**Supplementary Movie 3.** Timelapse of the *in vitro* melanization response in hemolymph in response to heat-killed *C. neoformans* and isolated fungal melanins.

**Supplementary Movie 4.** Timelapse of the *in vitro* melanization response in hemolymph in response to isolated fungal melanins*.*

**Supplementary Movie 5.** Timelapse of the *in vitro* melanization response in hemolymph in response to isolated fungal melanin from *C. neoformans.*

**Supplementary Movie 6.** Timelapse of the *in vitro* interactions between the *G. mellonella* hemocytes and isolated fungal melanins

**Supplementary Movie 7.** 3D Rendering of a fungal nodule within *G. mellonella* using different Z-stacked microscopy images.

**Supplementary Movie 8.** Timelapse of the *in vitro* melanization response in hemolymph in response to *C. albicans* yeast and hyphal/pseudohyphal forms.

**Supplementary Movie 9.** Examples of *C. albicans* surviving and growing following immune melanin-encapsulation during timelapse microscopy. The *C. albicans* then undergoes pseudohyphal or hyphal growth.

**Supplementary Movie 10.** Timelapse of the *in vitro* melanization response in hemolymph in response to *C. auris* yeast and pseudohyphal forms.

**Supplementary Movie 11.** Timelapse of hemocytes and hemolymph interacting with *C. neoformans* in an anticoagulation buffer to prevent coagulation and melanization.

**Supplementary Movie 12.** Timelapse of hemocytes and hemolymph with no fungus or fungal component added.
